# Exosome-Producing Follicle Associated Epithelium Is Not Involved in Uptake of PrPd from the Gut of Sheep (*Ovis aries*): An Ultrastructural Study

**DOI:** 10.1371/journal.pone.0022180

**Published:** 2011-07-18

**Authors:** Caroline Piercey Åkesson, Gillian McGovern, Mark P. Dagleish, Arild Espenes, Charles McL Press, Thor Landsverk, Martin Jeffrey

**Affiliations:** 1 Department of Basic Sciences and Aquatic Medicine, Norwegian School of Veterinary Science, Oslo, Norway; 2 Animal Health and Veterinary Laboratories Agency (AHVLA), Lasswade Laboratory, Penicuik, Midlothian, United Kingdom; 3 Department of Pathology, Moredun Research Institute, Penicuik, Midlothian, United Kingdom; Creighton University, United States of America

## Abstract

In natural or experimental oral scrapie infection of sheep, disease associated prion protein (PrP^d^) often first accumulates in Peyer's patch (PP) follicles. The route by which infectivity reaches the follicles is unknown, however, intestinal epithelial cells may participate in intestinal antigenic presentation by delivering exosomes as vehicles of luminal antigens. In a previous study using an intestinal loop model, following inoculation of scrapie brain homogenate, inoculum associated PrP^d^ was detected by light microscopy shortly (15 minutes to 3.5 hours) after inoculation in the villous lacteals and sub-mucosal lymphatics. No PrP^d^ was located within the follicle-associated epithelium (FAE), sub-FAE domes or the PP follicles. To evaluate this gut loop model and the transportation routes in more detail, we used electron microscopy (EM) to study intestinal tissues exposed to scrapie or control homogenates for 15 minutes to 10 days. In addition, immuno-EM was used to investigate whether exosomes produced in the FAE may possess small amounts of PrP^d^ that were not detectable by light microscopy. This study showed that the integrity of the intestinal epithelium was sustained in the intestinal loop model. Despite prominent transcytotic activity and exosome release from the FAE of the ileal PP in sheep, these structures were not associated with transportation of PrP^d^ across the mucosa. The study did not determine how infectivity reaches the follicles of PPs. The possibility that the infectious agent is transported across the FAE remains a possibility if it occurs in a form that is undetectable by the methods used in this study. Infectivity may also be transported via lymph to the blood and further to all other lymphoid tissues including the PP follicles, but the early presence of PrP^d^ in the PP follicles during scrapie infection argues against such a mechanism.

## Introduction

The oral route of infection is considered to be important in the natural pathogenesis of transmissible spongiform encephalopathies (TSEs), which are fatal neurodegenerative disorders described in man, domestic and wild ruminants and carnivores [1]. TSEs are characterised by the accumulation of an abnormal form of the host coded prion protein (PrP) in brain and some viscera [2]. The prion, or protein only hypothesis proposes that there are conformational variants of abnormal prion protein that encode strain specific information [3] but the nature of infectious prion protein remains uncharacterised. For diagnostic purposes disease associated forms of PrP (PrP^d^) may be detected by immunohistochemistry in tissue sections. The detection of PrP^d^ generally indicates the presence of infectivity, but the correlations between PrP^d^ and infectivity are not always precise as infectivity may be detected in the absence of PrP^d^ [4]–[6] and vice versa [7].

In natural TSE infections such as scrapie in sheep and various experimental animal models, the early accumulation of PrP^d^ occurs in the gut associated lymphoid tissues prior to clinical disease [8]–[10]. PrP^d^ has been detected in the Peyer's patch (PP) follicles as early as 21 days after inoculation in experimentally infected sheep [10]. The early presence of PrP^d^ in gut associated lymphoid tissues provides circumstantial evidence for uptake from the gut but does not give any direct insight into the route of intestinal transport of infection.

The mechanisms and route by which infectivity reaches the follicles after crossing the intestinal barrier remain unclear. The assumption is that PrP^d^ is taken up by the follicle associated epithelium (FAE) of PPs and transported across the sub-FAE dome region to enter the PP follicles, but direct evidence for this route of transmission is lacking. It is widely accepted that FAE, and particularly M cells, transport luminal antigens to subjacent antigen presenting cells as part of the maintenance of immune surveillance and oral tolerance, and that this physiological process is exploited as a route of entry for various pathogens [11]. Support for the notion that scrapie also uses this route of entry is provided by *in vivo* studies of rodents orally challenged with scrapie which show PrP^d^ within the FAE, possibly within M cells [12], [13], and *in vitro* studies which demonstrate transcytosis of prions within M cell cultures [14], [15]. In addition, dendritic cells have been implicated in transport of PrP^d^ or infectivity from the gut lumen to mesenteric lymph nodes [16] or via dome and to adjacent PP lymphoid tissues [17].

The intestinal transmission of scrapie in sheep has been studied in a gut loop model in which scrapie brain homogenates were incubated *in vivo* for various periods of time [18]. PrP^d^ positive material was found to be transported rapidly across the absorptive epithelium (AE) to reach the lacteals of the villi within 15 minutes of inoculation. PrP^d^ positive material was not detected along the presumed route of transmission, namely in the FAE, the domes and PP follicles during the initial 24 hours in which the inoculum was detectable. Nevertheless, when *de novo* generated PrP^d^ was detected after 30 days, it was present in the PP follicles. These observations invited speculation on the relationship between infectivity and immunohistochemically detectable PrP^d^. Thus, infectivity may have been carried across the FAE separately from PrP^d^. Alternatively, PrP^d^ and infectivity may have been carried to the PP at levels below the threshold of immunohistochemical detection.

Active macromolecular transport exists between the FAE and ileal PP follicles in cattle and sheep [19], [20]. Landsverk and co-workers have described the shedding of approximately 50 nm sized membrane-enveloped particles from multivesicular bodies (MVBs) in the FAE and traced these exosome-like vesicles from their formation in the luminal plasma membrane of the FAE to their apparent destination in the centre of the submucosal lymphoid follicles [19]. These *in vivo* observations correlate with the recent description of the release of prions in association with exosomes from sheep scrapie infected cultured cells [21]. The role of the FAE in macromolecular transport has been widely documented, but studies have also shown that the gut loop model can disturb these processes [22].

The object of the present study was to use electron microscopy (EM) to evaluate the gut loop model, especially the transcytotic activity in the FAE and transport of luminal material into the follicles, shown to occur under normal conditions in ileal PP of sheep [19], [23]. In addition, given the inability of Jeffrey *et al.* [18] to demonstrate uptake of PrP^d^ across the FAE, we undertook to determine whether immunogold-EM would reveal the presence of PrP^d^ in the PPs, and in particular whether exosomes could be carriers of PrP^d^.

## Results

### Electron microscopy (EM)

Generally, observations of all the intestinal tissues showed normal cell morphology, with normal nuclei and cellular organelles. The study of the intestine from loops exposed to scrapie infected brain inoculum, normal uninfected brain inoculum or sucrose-inoculum, did not reveal differences that could be attributed to the nature of the inoculum. Likewise, differences were not observed between surgically ligated/clamped loops and intestine not ligated or incised. However, variations between intestinal tissues sampled at the earliest time points (15 minutes to 3.5 hours) and later time points (24 hours to 10 days) were observed (Table 1 provides a semi-quantitative summary of the primary features seen at EM in PP follicular tissues taken at different time points).

**Table 1:** Showing the mean scores of selected observations in the ileal PP follicles according to the time of incubation and nature of inoculum.

Time points	Inocula	No samples	Intercellular space	Granular material	Vesicles	Electron dense material
15 m–3.5 h	Control	10	2.7	2.5	2.5	1.5
15m–2 h	Infected	7	2.7	2.7	2.7	1.2
24 h–10d	Control	6	1.5	1.3	1.8	1.7
24 h–10 d	Infected	5	2.0	1.6	2	1.8

A number of different morphological observations were found to change over time. Measurements were made of intercellular spaces between cells and of the intercellular content (granular material, vesicles and electron dense material) from intestinal loops challenged with infected (scrapie brain infected material inoculum) or control (normal brain or sucrose inoculum) material. The values are stated as mean values created by subjective assessments where 3 = large/abundant, 2 = moderate, 1 = narrow/sparse, and 0 = absent.

### Absorptive villous epithelium (AE)

**Figure 1:**
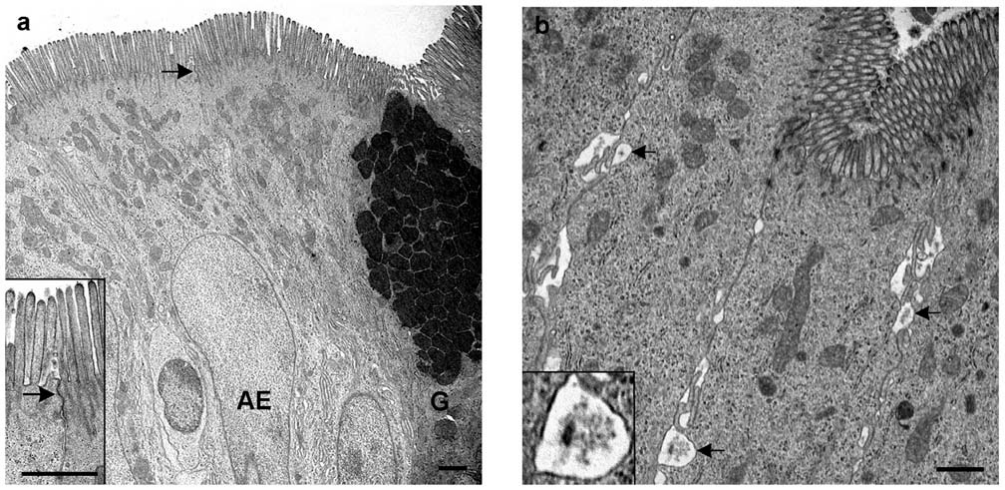
Ultrastructural observations of intestinal absorptive epithelium (AE) at early time points (15 minutes to 3.5 hours postinoculation). (a) AE showed close contact between each cell with a brush border composed of long regular and densely packed microvilli. Inset shows prominent microvilli and intact tight junctions (arrow). Goblet cells (G) were present among the AE cells. Branching tubular structures and MVBs were rare in the AE (animal R1292 2 hour sucrose loop). (b) Widened spaces (arrows) were present between occasional epithelial cells, and in a few of these a dark granular material was observed. Inset shows detail of intercellular granular material (animal R1292 15 minute infected loop). Bar, 1 µm.

**Figure 2:**
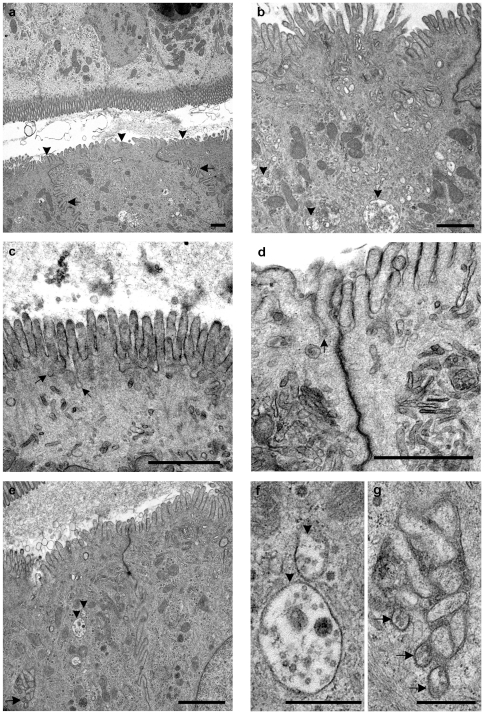
Ultrastructural observations of the follicle associated epithelium (FAE) at early time points (15 minutes to 3.5 hours postinoculation). (a, b, and e) 15 minutes to 2 hours post exposure to inoculum granular material, membranous whorls and vesicles were observed in the lumen. (a) The short surface projections of the FAE (arrowheads) contrast with the prominent brush border of the AE. The interdigitating lateral cell contacts of the FAE are also conspicuous (arrows). In contrast to the AE, FAE showed a high activity rate with many vesicles and branching tubular structures in the apical portion of the cells at early time points post inoculation (animal R1292 2 hour sucrose loop). (b) Higher magnification of FAE showing MVBs (arrowheads), vesicles and branching tubular structures in apical portions of the cell (animal R1525 1.5 hour control brain loop). (c) Deep pits (arrows) were formed in the apical cellular membrane of the FAE (animal R1294 15 minute infected loop). (d) Occasionally, these pits contained vesicles (arrows) (animal R1294 15 minute infected loop). (e, f, g) At two hours post inoculation, MVBs (arrowheads) (e and f) and exosome-like vesicles in intercellular lacunae (arrows) (e and g) were frequent (animal R1292 2 hour sucrose loop). Fig. 2a to e: Bar, 1 µm, Fig f and g: Bar, 0.5 µm.

The villi were covered with tall columnar absorptive cells presenting long regular and densely packed microvilli. Interspersed among these absorptive cells were goblet cells (Fig. 1a). Few vacuoles, tubulovesicular structures or MVBs were present in the cytoplasm of the AE of the villi in the loops from time points 15 minutes to 3.5 hours post exposure to inoculum. Moderately enlarged intercellular spaces of the AE were occasionally observed in the tip of the villi but tight junctions between the cells were intact. In one of the loops administered with scrapie infected brain inoculum, an amorphous granular material was observed in a few of these intercellular spaces (Fig. 1b). In the epithelial cells, vacuoles containing a few membrane-bounded vesicles corresponding to MVBs were observed infrequently, and the numbers of vacuolar and tubular structures including MVBs in the AE were much lower when compared with the apposing FAE (Fig. 2a). The epithelial cells of the intestinal loops exposed for 24 hours to 10 days were mainly in close contact, only a few slightly enlarged intercellular spaces without content were recognized in the tips of the villi (not shown).

### Follicle-associated epithelium (FAE)

The FAE showed the typical features previously reported [20] including short microvilli, which often showed coated vesicles at their base, interdigitations and indentations of the lateral cell border and numerous vesicles, vacuoles, dense and residual bodies (Fig. 2a and b). Many of the vacuoles could be categorized as MVBs, based on their contents of membrane-bounded particles or vesicles (compare [20]) that measured approximately 50 nm in diameter. At 15 minutes post exposure to inoculum, luminal material was present and diffusely distributed above many of the domes and close to the FAE. The luminal material consisted of a fibrillar or granular substance, membranous whorls, and vesicles (Fig. 2a, c and e). The vesicles varied in size, many being single membrane bounded and with a diameter between 30 to 80 nm. These were morphologically consistent with features of exosomes, and the term exosomes will hereby be used for the extracellular vesicles of this size range. The amount of luminal material decreased gradually and was distributed more multifocally with increasing incubation time. At 15 minutes to 3.5 hours post exposure to inoculum, many exosomes were in close contact with the FAE on the luminal side, both at the tip of and in between the microvilli, as well as appearing occasionally in pits formed in the luminal FAE cell membranes (Fig. 2c and d). The frequency of formation of deep pits in the apical cell membrane decreased with time, but was still seen intermittently, which seemed to correspond to the amount of luminal material in close contact with the FAE. Vacuoles, branching tubulovesicular structures, and MVBs were observed in the apical cytoplasm. These indicators of transcytotic activity were an increasing feature within the time span of 15 minutes to 3.5 hours (Fig. 2e, f, and g). In addition, numerous exosomes were present within intercellular indentations or lacunae of the FAE (Fig. 2e, and g).

Intestinal tissues exposed for 24 hours to 10 days showed some variation between loops and between domes within each time point, but in general there was very little or no evidence of luminal material covering the FAE of the domes. The FAE showed low transcytotic activity, as judged by the observations of few vacuoles, branching tubular structures and MVBs in the apex of the cells at these time points. In addition, very few exosomes were observed in intercellular lacunae (data not shown).

### Sub-FAE domes

**Figure 3:**
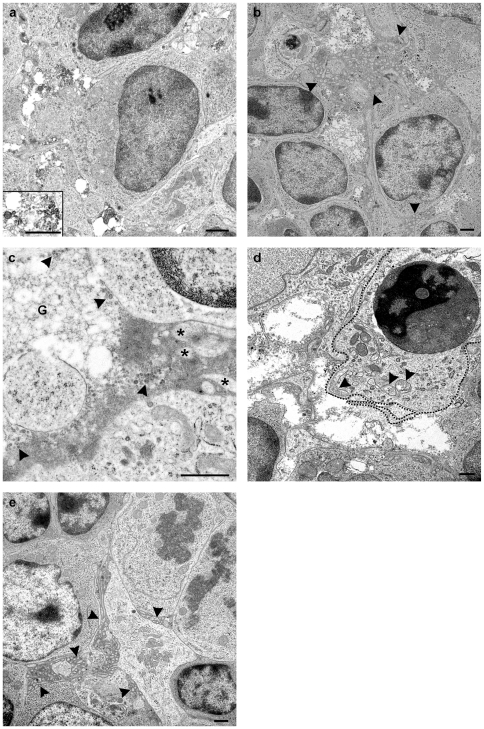
Ultrastructural observations of domes and follicles. (a) In domes, extracellular vesicles of heterogenous appearance including exosomes were present together with granular material. Inset shows higher magnification of intercellular content (animal R1292 15 minute sucrose loop). (b) Intercellular spaces within the PP follicle were widened at early time points (15 minute to 3.5 hours) post inoculation. These spaces contained granular material and heterogenous appearing vesicles, many resembling exosomes, in addition to amorphous electron dense material surrounding FDC dendrites (arrowheads) (animal R1292 2 hour infected loop). (c) Detail of amorphous electron dense material surrounding FDC dendrites (*), granular material (G), and exosome-like vesicles (arrowheads) were abundant (animal R1292 2 hour infected loop). (d) In the PP follicles at early time points, many macrophages (delineated) were observed in the process of phagocytosing (arrowheads) extracellular material (animal R1520 3.5 hour control brain loop). (e) At time points 24 hours and onwards, the intercellular spaces had narrowed, and the amount of granular material and exosomes had decreased. FDC dendrites were observed between cells, surrounded by electron dense material (arrowheads). In addition, uptake of extracellular material by macrophages observed in the early time points could not be seen after 24 h (animal R1272 24 hour infected loop). Bar, 1 µm.

Exosomes were variably present in the sub-FAE dome. At 15 minutes to 3.5 hours post exposure, large numbers of heterogeneous appearing vesicles, including exosomes (30–80 nm), showed a multifocal distribution in intercellular spaces (Fig. 3a). At time points 24 hours to10 days, vesicles and particles were generally absent in the sub-FAE dome (data not shown).

### Lymphoid follicles

Material from loops taken 15 minutes to 3.5 hours post-exposure to inoculum showed wide spaces between the cells in the central zone of the PP follicles. These spaces contained large amounts of granular material and vesicles, in addition to a sparse electron dense amorphous material surrounding the follicular dendritic cell (FDC) dendrites (Fig. 3b and c). The vesicles were heterogeneous with respect to form, density and size-range. Exosomes of dimensions and appearance consistent with those found in the FAE and dome were also observed in the PP follicles. At the time points 15 minutes to 3.5 hours post-exposure to inoculum many macrophages were shown to have multiple cytoplasmic vacuoles, which were close to the surface of the cell and enclosed the same material observed in the extracellular spaces. This observation was interpreted to indicate uptake of this material by macrophages (Fig. 3d). From 24 hours and onwards this type of phagocytic activity in macrophages was not observed. In addition, the amount of granular material and exosomes decreased to a sparse or moderate amount, while the electron dense amorphous material increased to a moderate amount surrounding FDC dendrites (Fig. 3e). (See table 1 for summary of features)

### Immunogold: PrP^d^ and GFAP

**Figure 4:**
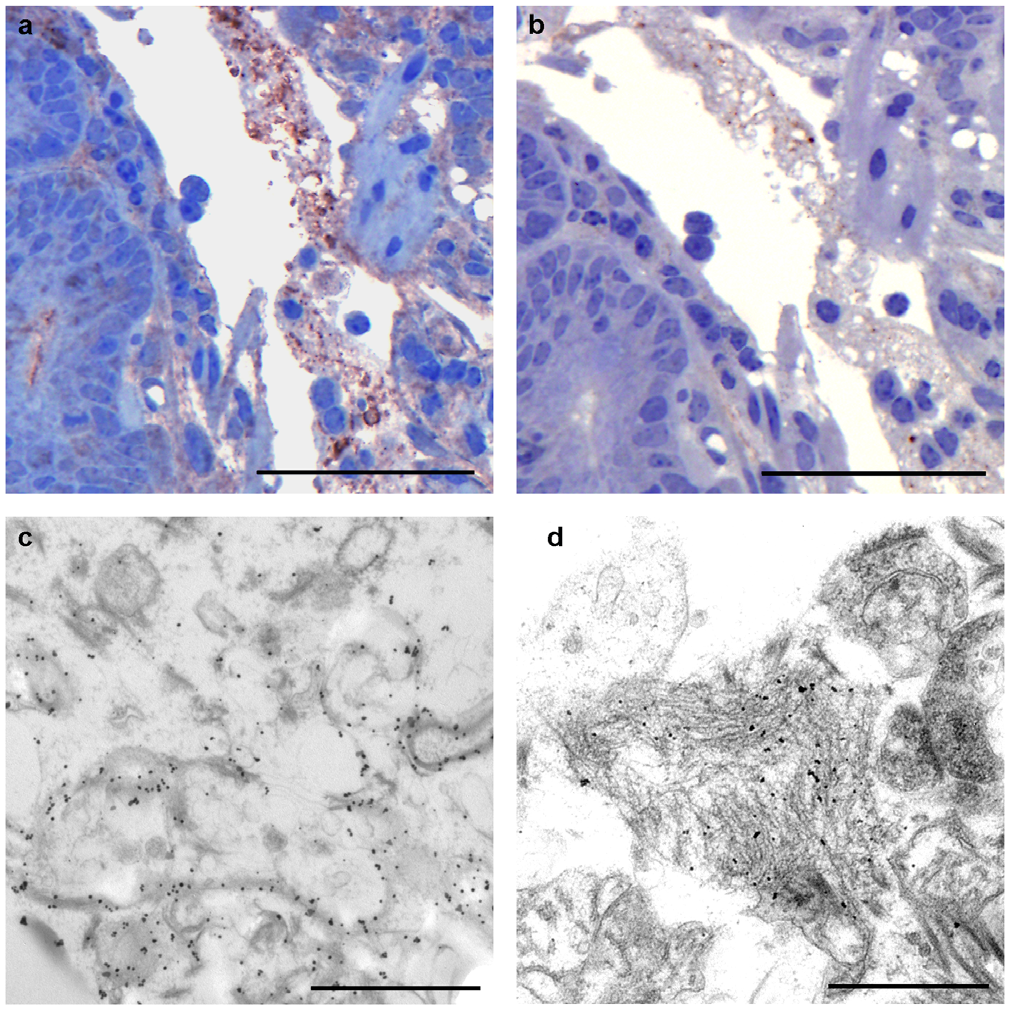
PrP- and GFAP- labelling on lacteal material. Immunohistochemistry showing positive labelling for PrP (a) and GFAP (b) on granular material present in villous lacteal of intestinal loop inoculated with scrapie brain inoculum for 15 minutes (animal R1293). Bar, 50 µm. Taking these samples further to immuno-EM-labelling, revealed PrP- (c) and GFAP-labelling (d) on fibrillar material in the villous lacteal. Bar, 0.5 µm.

Amorphous material was observed in lacteals of some villi of intestinal loops exposed to both scrapie brain inoculum (9 villi in total) and sucrose loops (3 villi in total) at early time points (15 minutes to 2 hours). These sections with villi lacteals containing amorphous material were labelled with antibodies against PrP^d^ and GFAP (Fig. 4a and b, respectively). Positive labelling for both PrP^d^ and GFAP was observed in two infected 15 minute loops only, while villous lacteal material was negative in all sucrose inoculated loops. Following EM immunogold labelling, PrP^d^ and GFAP-positive labelling was observed in the same sections that were positive at light microscopy (Fig. 4c and d, respectively). FAE, domes and follicles from 15 minute and 2 hour loop material from ileal PP did not show immunogold labelling for PrP^d^ at EM level (not shown). Control animals had a low grade level of gold particles dispersed with no concentration at any specific localisation (not shown).

## Discussion

The experimental intestinal loop model enables *in vivo* events occurring in the intestinal wall at specified times after exposure of the gut to a particular agent or substance to be studied [23]. Jeffrey *et al.* [18] used the intestinal loop model to follow the uptake and distribution of a scrapie inoculum beyond the epithelial layer and into different intestinal compartments. The inoculum derived PrP^d^ was detectable in the intestinal wall for 24 hours following exposure but contrary to expectation it was present in lacteals of the villi and not in the dome and follicles of the PP. One explanation for the absence of uptake of scrapie inoculum across the FAE was that the experimental procedure had disturbed the function and/or structure of the intestinal epithelium. As surgical trauma may induce loss of the macromolecular barrier in the intestine, the importance of caution in the interpretation of loop experiments was pointed out by Rhodes et al [22]. The present ultrastructural investigation of the loop material discounts this explanation.

**Figure 5:**
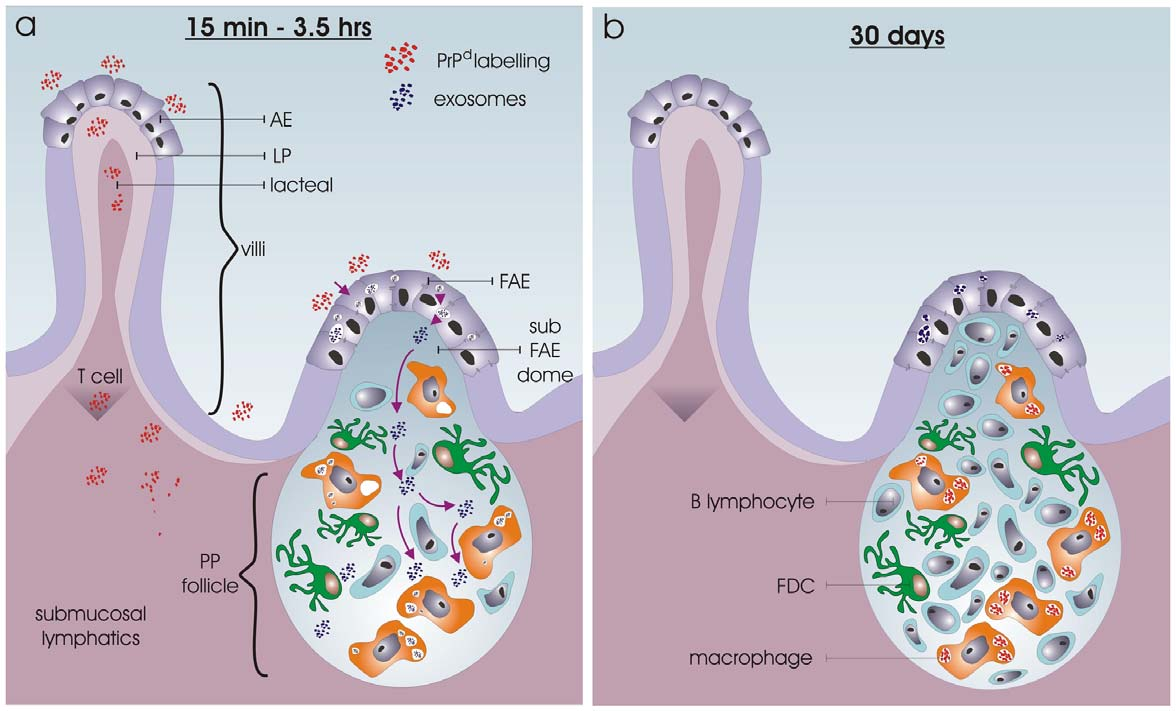
Diagram showing EM observations of early scrapie pathogenesis after intestinal infection (a), and corresponding light microscopical findings of Jeffrey *et al.* at 30 days post infection (b) [**18**]. 5a. PrP^d^ detectable material is transported across the villi into the lacteal and submucosal lymphatics. Despite EM evident transcytotic activity in the FAE, exosome production and transport to the PP follicle, in addition to uptake of extracellular material by germinal centre macrophages, PrP^d^ is not detectable in these intestinal structures. 5b. *De novo* generated PrP^d^ is initially observed in macrophages at 30 days post infection (*Jeffrey et a.l* [18]), however the route of infection remains to be elucidated.

Ultrastructural evidence of transcytosis was present in the FAE including pitting of the luminal membrane and the presence of many vacuoles, tubulovesicular structures and MVBs in the apical cytoplasm. The evidence of uptake of extracellular material in macrophages also argues for transcytosis by the FAE and transport of luminal material into the follicles as shown in the ileal PP of lambs [19], [24]. The structure and integrity of the intestinal tissues, including the presence of intact epithelial tight junctions, indicate a physiological trans-epithelial transport of PrP^d^ across an intact AE, and a concurrent exclusion of PrP^d^ from a functional FAE, as depicted in Figure 5a.

Another strong indication of a functional FAE in the isolated intestinal loop was the presence of abundant exosomes in the domes and follicles. Landsverk *et al.* [19], [24] found that the FAE of sheep ileum shed numerous ∼50 nm membrane-bounded particles into the intercellular spaces of FAE and documented the transport of these structures into the lymphoid follicles of the PP. The formation of the ∼50 nm particles by budding from the membrane of MVBs, their shedding by exocytosis and resistance against detergents suggest that these ∼50 nm particles belong to the exosome family [25]. Exosomes have previously been implicated in scrapie pathogenesis. Fevrier and co-workers [21] found that PK resistant PrP and infectivity were released in association with exosomes by infected epithelial cells, and suggested that this could provide an acellular mechanism underlying the spread of infectivity. Whether exosomes released from FAE transfer infectivity has not been studied but it has been suggested that FAE is involved in the uptake and transport of infectivity [13], [26]. FAE and M cell involvement in prion transport was given added credence by the conclusions of Heppner *et al.* [14] that M cell-differentiation was necessary for active transepithelial transport, and that transcytosis does not occur through Caco-2 epithelial cell cultures lacking M cells.

Given the demonstration that M cells can transcytose prions both *in vitro* [14] and *in vivo* [13], in addition to the known production of exosomes by the FAE, the present study examined whether exosomes shed by the FAE carried PrP^d^. Immunohistochemistry and light microscopy of intestinal wall compartments known to harbour exosomes did not reveal the presence of PrP^d^ in FAE, dome or lymphoid follicles of isolated intestinal loops exposed to scrapie-infected brain material [18]. Nevertheless, the possibility of a low level of PrP^d^ on exosomes below the detection limit of light microscopy that could be detected by immunogold labelling and EM had not been ruled out. The present study tested this hypothesis but found that the exosomes were PrP^d^-negative (Figure 5a). An ultrastructural study of mesenteric lymph nodes from sheep showing clinical disease following natural scrapie infection [27] demonstrated exosomes in the extracellular spaces between the dendrites of some scrapie-affected FDCs. As with the present findings, these investigators found no evidence that exosomes in PrP^d^ labelled secondary follicles carry PrP^d^.

The notion of FAE involvement in prion transport is apparently in conflict with the *in vivo* findings reported by Jeffrey *et al.* [18] and the present study. Both these studies show that PrP^d^ was located in villous lacteals and not in or associated with the FAE. However, the result could be explained by the studies of Landsverk *et al.* [28], [29], which show that FAE of the ileal PP in lambs lacks conventional M cells. Although the ileal FAE shows many similarities to the M cell, such as abundant transcytotic activity, the ileal FAE, in contrast to M cells, contains acid phosphatase rich vacuoles and dense bodies in the apical cytoplasm. At the level of the FAE, there is a sorting of endocytosed material into such compartments destined either for transcytosis or for lysosomes/residual bodies [30]. These findings imply that PrP^d^ taken up into the ileal FAE could be degraded completely or partially into aggregates undetectable by our methods. Moreover, any PrP^d^ released into the sub-epithelial spaces of the dome could be scavenged by the rim of macrophages present at this site [31].

In addition to the possibility of degradation in the FAE, degradation processes apparently affecting PrP^d^ might occur in the intestinal contents. Jeffrey *et al.* [18] showed that PrP^d^ was rapidly digested by alimentary fluids, hence it cannot be excluded that PrP^d^ was degraded into components undetectable by anti-PrP antibodies used in the present study. However, this explanation would require a selective degradation in the FAE, dome and follicle that was not operative or at least less prominent in the AE of villi as PrP^d^ labelled material was present in the villi lacteals.

Uptake and transport of material from the gut lumen to the intestinal compartments is a rapid and dynamic process [32]. We cannot exclude the possibility that uptake of PrP^d^ may have occurred in the FAE at time points that were not examined. Nevertheless, studies in ruminants [30], [33] and other species [34], [35] indicate that transport across the FAE would be expected to occur during the time spans investigated in the present study. Also, if PrP^d^ had been transported across the FAE to the follicle centre earlier than at the time points investigated, a reasonable assumption would be that PrP^d^ should have accumulated in the phagocytosing macrophages and be detectable in lysosomes, as has been demonstrated to occur shortly after culturing macrophage cell lines with scrapie infected brain material [36], at early and late stages of experimental murine and sheep scrapie [37] (Fig. 5b), and naturally diseased and clinically affected sheep [27]. However, despite that the present study found that exosomes and granular material were transported from the FAE and domes and into the follicles where the material appeared to accumulate extracellularly and to be taken up by macrophages, no immunogold detectable PrP^d^ were transported into the follicles. Thus, the mechanisms by which infectivity rapidly reached the follicles of PPs 30 days after inoculation of the loops, as demonstrated by the accumulation of *de novo* generate PrP^d^ [18], remains undetermined. This is illustrated in Figure 5.

It is possible that infectivity could be transported across the villi to the lymph then to regional lymph nodes and back to the PP follicle via the blood. The presence of TSE infectivity in blood is established, as BSE has been shown to be transmitted from orally infected donors to recipients after a single blood transfusion [38], [39]. Similarly, the infectivity of the blood from sheep affected with natural scrapie has been demonstrated by blood transfusion to sheep recipients [40]. The hypothesis that infectivity may be disseminated by the vascular system can be further strengthened by the recent findings by Sisó *et al.* [41], who demonstrated that infection is transported to the brain by the haematogenous route, regardless of how infection enters the body. However, haematogenous dissemination of infectivity would imply equal exposure of and subsequent simultaneous appearance of PrP^d^ in all lymphoid tissues (and non-lymphoid tissues). In naturally or orally infected animals, numerous studies have reported that PrP^d^ accumulation appears initially in lymphoid follicles of PPs followed by accumulation in other lymphoid tissues at later stages [8]–[10]. This consistent finding argues for a local effect.

## Materials and Methods

### Animals and surgical procedures

The animal experiments carried out within VLA were supervised by a named Veterinary surgeon as required under UK legislation and were approved by UK government Home office inspectors; approval ID E04 50.

Details of surgical methods [42] and experiment animals [18] have previously been published. Briefly, New Zealand derived, scrapie-free lambs of the PrP^ARQ/ARQ^ genotype were weaned at two months of age, and then starved, anaesthetised and a midline laparotomy performed. The distal ileum with its continuous PP was formed into a large loop by sectioning the intestine proximally and distally. The isolated segment was flushed with saline containing antibiotics and its ends were either closed or clamped, depending on whether the lamb was to be allowed to recover from anaesthesia or was scheduled for loop removal at an early time point (see below). The remaining bowel was re-anastomosed or clamped. The large isolated loop was then sub-divided into two or four smaller loops by ligation. In each lamb, alternate loops were inoculated with 10 ml (equivalent to 0.5 g) of a scrapie brain pool inoculum or with either normal brain homogenate or with sucrose solution (Table 2). In a second strategy the gut loop of three lambs was exteriorised and inoculated with sucrose, normal sheep brain or scrapie brain homogenate but no further procedures were performed; the homogenate was retained in position solely by the effect of gravity. Some dilution of the homogenate occurred with existing intestinal contents. As no significant differences were found between exteriorised and ligated loops or between sucrose, infected and normal brain loops the results below do not distinguish between surgical methods or different kinds of control inocula.

**Table 2:** Table showing ID and numbers of sheep in which the loops were created, the time points at which tissues were exposed to inoculum, and numbers of samples studied from each loop.

	Inocula (number of samples studied from each loop)			
Animal ID	Time points	Scrapie infected brain	Control sucrose	Control normal
R1292	15 min	3*	2	
R1293	15 min	2*	2*	
R1294	15 min	2	1	
R1525	1.5 h			2†
R1292	2 h	2	2	
R1294	2 h	1	1	
R1518	3.5 h			2† *
R1520	3.5 h			1†
R1272	24 hr	2	2	
R1287	24 hr	1	1	
R1273	3 d	2*	1	
R1274	5 d	1*	1	
R1289	10 d	1	1	

† Intestinal samples not flushed and not surgically ligated or incised.

*Loop was sampled but in one or more of these samples PP follicular tissue was not studied and therefore not represented in the results listed in Table 1.

From the above strategies, loops were removed from a total of six lambs at time points after inoculation ranging from 15 minutes to 3.5 hours (Table 2), without allowing the lambs to recover from anaesthesia. The remaining five lambs were allowed to recover from anaesthesia after the loops were inoculated and were killed at one, three, five, and ten days post challenge.

### Inocula

Brains from 10 scrapie-confirmed clinically affected ARQ/ARQ sheep were obtained from an intensely studied Suffolk flock [43]. These brains were pooled and homogenised to form a 1/5 stock, which was further diluted in 0.32 M sucrose solution to a final 10^−1^ dilution for inoculation of the gut loops. A normal brain homogenate from unaffected sheep was prepared in the same way for inoculation into the control loops. The oral and subcutaneous infectivity of this inoculum for sheep of different genotypes has been established in different experiments (unpublished data).

### Histopathology, immunohistochemistry and electron microscopy

A wide range of visceral and CNS tissues were sampled at necropsy and light microscopy results of these tissues have previously been published [18]. Only small intestine containing PP are examined here. For the present study samples of intestinal loops were fixed in formalin, embedded in paraffin wax and stained by haematoxylin and eosin or labelled for PrP using antibodies 1A8 [44] and R486 (R. Jackman, VLA, Weybridge, UK). For EM samples of distal ileum containing PP were immediately removed at necropsy and 1 mm wide strips across the PP were fixed in 0.5% glutaraldehyde and 4% paraformaldehyde, post fixed in 1% osmium tetroxide. These 1 mm wide strips of PPs were further sectioned to 1 mm cubes and routinely processed to araldite (Taab laboratories, Berks, UK). Resin embedded sections were cut at 1 µm and stained with toluidine blue or labelled with the PrP antibodies 1A8 [29], or 523.7 (J. Langeveld, ID–Lelystad, Netherlands) [45], [46] or for GFAP [47] as previously described. Resin blocks containing representation of follicles, domes and villi from each loop of each sheep were then sectioned at 60 nm and routinely stained using uranyl acetate and lead citrate. Sections were then immunolabelled for PrP^d^ using the above antibodies by immunogold methods as previously described [45]. Positive control material used in immunogold EM included brain from a confirmed scrapie-positive animal containing abundant PrP^d^ and GFAP.

To ascertain whether there were any morphological features which might be attributed to early scrapie infection, grids from each loop were coded and examined blind. Features present in each section were listed and each feature was subjectively assessed as large/abundant, moderate, narrow/sparse or absent. To compare effects of scrapie challenge with the controls these categories were converted to scores of 3-0 (Table 2).

## References

[1] Hadlow WJ, Kennedy RC, Race RE (1982). Natural infection of Suffolk sheep with scrapie virus.. J Infect Dis.

[2] Jeffrey M, González L, Harris D (2004). Pathology and pathogenesis of bovine spongiform encephalopathy and scrapie.. Mad Cow Disease and Related Spongiform Encephalopathies.

[3] Prusiner SB, Pulendran B (1999). Development of the prion concept.. Prion Biology and Diseases.

[4] Barron RM, Campbell SL, King D, Bellon A, Chapman KE (2007). High titers of transmissible spongiform encephalopathy infectivity associated with extremely low levels of PrPSc in vivo.. J Biol Chem.

[5] Lasmezas CI, Deslys JP, Robain O, Jaegly A, Beringue V (1997). Transmission of the BSE agent to mice in the absence of detectable abnormal prion protein.. Science.

[6] Race R, Meade-White K, Raines A, Raymond GJ, Caughey B (2002). Subclinical scrapie infection in a resistant species: persistence, replication, and adaptation of infectivity during four passages.. J Infect Dis.

[7] Piccardo P, Manson JC, King D, Ghetti B, Barron RM (2007). Accumulation of prion protein in the brain that is not associated with transmissible disease.. Proc Natl Acad Sci U S A.

[8] Andréoletti O, Berthon P, Marc D, Sarradin P, Grosclaude J (2000). Early accumulation of PrP(Sc) in gut-associated lymphoid and nervous tissues of susceptible sheep from a Romanov flock with natural scrapie.. J Gen Virol.

[9] van Keulen LJ, Vromans ME, van Zijderveld FG (2002). Early and late pathogenesis of natural scrapie infection in sheep.. APMIS.

[10] Ryder SJ, Dexter GE, Heasman L, Warner R, Moore SJ (2009). Accumulation and dissemination of prion protein in experimental sheep scrapie in the natural host.. BMC Vet Res.

[11] Corr SC, Gahan CC, Hill C (2008). M-cells: origin, morphology and role in mucosal immunity and microbial pathogenesis.. FEMS Immunol Med Microbiol.

[12] Beekes M, McBride PA (2000). Early accumulation of pathological PrP in the enteric nervous system and gut-associated lymphoid tissue of hamsters orally infected with scrapie.. Neurosci Lett.

[13] Foster N, MacPherson GG (2010). Murine cecal patch M cells transport infectious prions in vivo.. J Infect Dis.

[14] Heppner FL, Christ AD, Klein MA, Prinz M, Fried M (2001). Transepithelial prion transport by M cells.. Nat Med.

[15] Ghosh S (2004). Mechanism of intestinal entry of infectious prion protein in the pathogenesis of variant Creutzfeldt-Jakob disease.. Adv Drug Deliv Rev.

[16] Huang FP, Farquhar CF, Mabbott NA, Bruce ME, MacPherson GG (2002). Migrating intestinal dendritic cells transport PrP(Sc) from the gut.. J Gen Virol.

[17] Defaweux V, Dorban G, Demonceau C, Piret J, Jolois O (2005). Interfaces between dendritic cells, other immune cells, and nerve fibres in mouse peyer's patches: potential sites for neuroinvasion in prion diseases.. Microscopy Research and Technique.

[18] Jeffrey M, González L, Espenes A, Press CM, Martin S (2006). Transportation of prion protein across the intestinal mucosa of scrapie-susceptible and scrapie-resistant sheep.. J Pathol.

[19] Landsverk T, Jansson A, Nicander L, Plöen L (1987). Carbonic anhydrase–a marker for particles shed from the epithelium to the lymphoid follicles of the ileal Peyer's patch in goat kids and lambs.. Immunol Cell Biol.

[20] Landsverk T (1987). The follicle-associated epithelium of the ileal Peyer's patch in ruminants is distinguished by its shedding of 50 nm particles.. Immunol Cell Biol.

[21] Fevrier B, Vilette D, Archer F, Loew D, Faigle W (2004). Cells release prions in association with exosomes.. Proc Natl Acad Sci U S A.

[22] Rhodes RS, Karnovsky MJ (1971). Loss of macromolecular barrier function associated with surgical trauma to the intestine.. Lab Invest.

[23] Gerdts V, Uwiera RR, Mutwiri GK, Wilson DJ, Bowersock T (2001). Multiple intestinal ‘loops’ provide an in vivo model to analyse multiple mucosal immune responses.. J Immunol Methods.

[24] Landsverk T, Trevella W, Nicander L (1990). Transfer of carbonic anhydrase-positive particles from the follicle-associated epithelium to lymphocytes of Peyer's patches in foetal sheep and lambs.. Cell Tissue Res.

[25] Collas P, Cline R, Landsverk HB, Hein WR, Goldsby RA (2002). DNA-containing extracellular 50-nm particles in the ileal Peyer's patch of sheep.. Eur J Cell Biol.

[26] Heggebø R, Press CM, Gunnes G, Lie KI, Tranulis MA (2000). Distribution of prion protein in the ileal Peyer's patch of scrapie-free lambs and lambs naturally and experimentally exposed to the scrapie agent.. J Gen Virol.

[27] McGovern G, Jeffrey M (2007). Scrapie-specific pathology of sheep lymphoid tissues.. PLoS One.

[28] Landsverk T (1981). The epithelium covering Peyer's patches in young milk-fed calves. An ultrastructural and enzyme histochemical investigation.. Acta Vet Scand.

[29] Landsverk T, Halleraker M, Aleksandersen M, McClure S, Hein W (1991). The intestinal habitat for organized lymphoid tissues in ruminants; comparative aspects of structure, function and development.. Veterinary Immunology and Immunopathology.

[30] Landsverk T (1988). Phagocytosis and transcytosis by the follicle-associated epithelium of the ileal Peyer's patch in calves.. Immunol Cell Biol.

[31] Halleraker M, Landsverk T, Nicander L (1990). Organization of ruminant Peyer's patches as seen with enzyme histochemical markers of stromal and accessory cells.. Vet Immunol Immunopathol.

[32] Hazzard RA, Hodges GM, Scott JD, McGuinness CB, Carr KE (1996). Early intestinal microparticle uptake in the rat.. J Anat.

[33] Paar M, Liebler EM, Pohlenz JF (1992). Uptake of Ferritin by Follicle-associated Epithelium in the Colon of Calves.. Veterinary Pathology.

[34] Wolf JL, Rubin DH, Finberg R, Kauffman RS, Sharpe AH (1981). Intestinal M cells: a pathway for entry of reovirus into the host.. Science.

[35] Owen RL (1999). Uptake and transport of intestinal macromolecules and microorganisms by M cells in Peyer's patches–a personal and historical perspective.. Semin Immunol.

[36] Sassa Y, Inoshima Y, Ishiguro N (2010). Bovine macrophage degradation of scrapie and BSE PrPSc.. Vet Immunol Immunopathol.

[37] Jeffrey M, McGovern G, Martin S, Goodsir CM, Brown KL (2000). Cellular and sub-cellular localisation of PrP in the lymphoreticular system of mice and sheep.. Arch Virol.

[38] Houston F, Foster JD, Chong A, Hunter N, Bostock CJ (2000). Transmission of BSE by blood transfusion in sheep.. Lancet.

[39] Hunter N, Foster J, Chong A, McCutcheon S, Parnham D (2002). Transmission of prion diseases by blood transfusion.. J Gen Virol.

[40] Houston F, McCutcheon S, Goldmann W, Chong A, Foster J (2008). Prion diseases are efficiently transmitted by blood transfusion in sheep.. Blood.

[41] Sisó S, Jeffrey M, González L (2009). Neuroinvasion in sheep transmissible spongiform encephalopathies: the role of the haematogenous route.. Neuropathol Appl Neurobiol.

[42] Eaton SL, Anderson MJ, Hamilton S, González L, Sales J (2009). CD21 B cell populations are altered following subcutaneous scrapie inoculation in sheep.. Vet Immunol Immunopathol.

[43] Jeffrey M, Martin S, Thomson JR, Dingwall WS, Begara-McGorum I (2001). Onset and distribution of tissue prp accumulation in scrapie-affected suffolk sheep as demonstrated by sequential necropsies and tonsillar biopsies.. J Comp Pathol.

[44] Farquhar CF, Somerville RA, Doman J, Armstrong D, Birkett C (1993). A review of the detection of PrP^Sc^.. Transmissible Spongiform Encephalopathies, Proceedings of a consultation on BSE with the scientific veterinary committee of the commision of the European Communities.

[45] McGovern G, Brown KL, Bruce ME, Jeffrey M (2004). Murine scrapie infection causes an abnormal germinal centre reaction in the spleen.. J Comp Pathol.

[46] Jeffrey M, McGovern G, Goodsir CM, Sisó S, González L (2009). Strain-associated variations in abnormal PrP trafficking of sheep scrapie.. Brain Pathol.

[47] Jeffrey M, Goodsir CM, Race RE, Chesebro B (2004). Scrapie-specific neuronal lesions are independent of neuronal PrP expression.. Ann Neurol.

